# Monitoring Genomic Sequences during SELEX Using High-Throughput Sequencing: Neutral SELEX

**DOI:** 10.1371/journal.pone.0009169

**Published:** 2010-02-11

**Authors:** Bob Zimmermann, Tanja Gesell, Doris Chen, Christina Lorenz, Renée Schroeder

**Affiliations:** 1 Max F. Perutz Laboratories, Department of Biochemistry, University of Vienna, Vienna, Austria; 2 Center for Integrative Bioinformatics in Vienna, Max F. Perutz Laboratories, University of Vienna, Medical University of Vienna and University of Veterinary Medicine, Vienna, Austria; Centre de Regulació Genòmica, Spain

## Abstract

**Background:**

SELEX is a well established *in vitro* selection tool to analyze the structure of ligand-binding nucleic acid sequences called aptamers. Genomic SELEX transforms SELEX into a tool to discover novel, genomically encoded RNA or DNA sequences binding a ligand of interest, called genomic aptamers. Concerns have been raised regarding requirements imposed on RNA sequences undergoing SELEX selection.

**Methodology/Principal Findings:**

To evaluate SELEX and assess the extent of these effects, we designed and performed a Neutral SELEX experiment omitting the selection step, such that the sequences are under the sole selective pressure of SELEX's amplification steps. Using high-throughput sequencing, we obtained thousands of full-length sequences from the initial genomic library and the pools after each of the 10 rounds of Neutral SELEX. We compared these to sequences obtained from a Genomic SELEX experiment deriving from the same initial library, but screening for RNAs binding with high affinity to the *E. coli* regulator protein Hfq. With each round of Neutral SELEX, sequences became less stable and changed in nucleotide content, but no sequences were enriched. In contrast, we detected substantial enrichment in the Hfq-selected set with enriched sequences having structural stability similar to the neutral sequences but with significantly different nucleotide selection.

**Conclusions/Significance:**

Our data indicate that positive selection in SELEX acts independently of the neutral selective requirements imposed on the sequences. We conclude that Genomic SELEX, when combined with high-throughput sequencing of positively and neutrally selected pools, as well as the gnomic library, is a powerful method to identify genomic aptamers.

## Introduction

**Figure 1:**
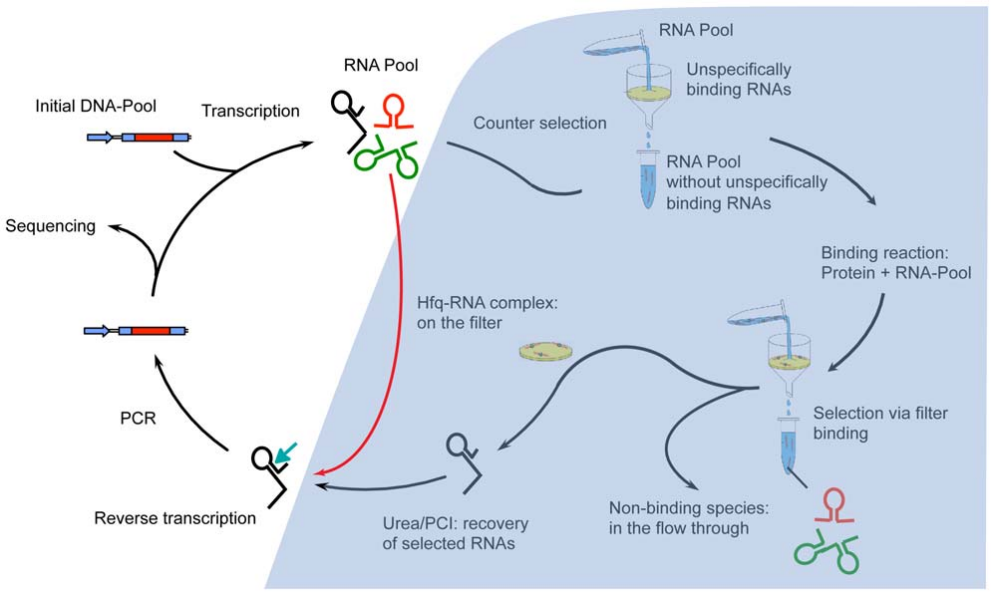
The SELEX and Neutral SELEX experiments. The normal SELEX experiment (shown with black arrows) begins with transcription of a DNA pool into RNA using T7 RNA polymerase. The RNAs then undergo counter selection against sequences binding non-specifically to the filter, followed by binding to the protein of choice, filtration and extraction. In order to amplify the sequences, the RNAs are reverse-transcribed and PCR-amplified, which generates the pool for the next cycle. Neutral SELEX (shown with the red arrow) bypasses the binding and filtration steps and the cycle is reduced to the amplification steps of the SELEX cycle.

Systematic Evolution of Ligands by EXponential enrichment (SELEX) is an *in vitro* strategy to analyze RNA sequences that perform an activity of interest, most commonly high affinity binding to a ligand [1]. The screen begins with an initial heterogeneous pool of random sequences. A successful RNA SELEX experiment reduces this pool to a more homogenized population of RNA sequences exhibiting binding activity. This is achieved through rounds of selection followed by amplification (Figure 1, black arrows). SELEX has been used widely, to study properties of RNA or DNA molecules that bind to proteins, small molecules, oligonucleotides and peptides among others [2]. Where SELEX was primarily used as a tool to analyze the structure of RNA aptamers to a given ligand, Genomic SELEX transforms the method into a discovery tool to screen endogenous RNAs by starting from a pool derived by random priming of the genome of interest. The *in vitro* nature of Genomic SELEX as a detector of functional RNA sequences circumvents the expression-level bias of co-immunoprecipitation and other *in vivo* strategies, and also does not require a prior model, as *in silico* methods do [3].

With the development of massive sequencing techniques and the availability of genome sequences, Genomic SELEX has the potential to be used as a very powerful selection procedure to discover genomic aptamers, i.e. sequences within RNAs that bind ligands with high affinity and are thus able to act as sensors or receptors within regulatory domains. The binding domain of riboswitches is a typical example of a genomic aptamer, which senses the concentration of metabolites.

Various researchers have suggested that functional RNAs have a more stable (lower minimum free energy) secondary structure than expected by chance, since most known functional RNAs depend on a defined secondary structure [4], [5], [6]. Many modern *in silico* methods indeed measure structural stability as partial evidence of a functional RNA [7], [8], [9], [10]. Thus, one might assume that since RNA sequences from a SELEX experiment are candidates for functional RNAs, that these sequences would exhibit the same feature. Yet, in a survey comparing sequences artificially selected with SELEX to naturally selected RNA sequences, it was observed that the artificially selected RNAs generally, but not necessarily, had predicted secondary structures that were less stable on average than naturally selected molecules [11]. The authors argue that the differing principles of artificial and natural selection are responsible for this phenomenon, but also speculate that the amplification steps could be causal. Since the analysis was carried out only on artificially selected sequences, the question of whether the non-selection steps themselves play a consequential role remained open.

We decided to explore the extent to which the SELEX selection procedure, independent of selection for the activity of interest, can influence the structural stability and other features of resulting sequences. We used an *E. coli* genomic library generated for the selection of RNA sequences with high affinity to the *E. coli* protein Hfq [12] to perform a “Neutral” SELEX experiment. The Hfq Genomic SELEX experiment included a step of binding with regulatory protein Hfq, followed by filtration and recovery of RNAs for amplification, whereas in the Neutral SELEX experiment, all selection steps are bypassed such that the sequences undergo no selection for activity, and changes in the sequence characteristics result from the neutral forces in the SELEX amplification steps only (Figure 1, red arrow). The pools obtained after each of the 10 rounds of Neutral SELEX were subjected to *454* sequencing.

We then compared these data with the sequences of the positive selection for high-affinity Hfq-binding RNAs mentioned above, which were sequenced alongside the Neutral SELEX pools. With this, we show that the sequences under SELEX constraints shift toward less stable predicted secondary structures and change slightly in nucleotide content with each successive round of Neutral SELEX. However, the sequences from the Hfq Genomic SELEX pool were highly enriched genomic aptamers, whereas in the Neutral SELEX pool, only background levels of enrichment could be detected. The mono- and dinucleotide content of sequences in Neutral and Hfq Genomic SELEX pools also differed significantly. Taking our data and analyses into account, we conclude that with sufficient sequencing and bioinformatic analysis, Genomic SELEX can be a potent method for enriching genomic aptamers.

## Results

### Neutral SELEX Experiment

To follow the evolution of RNA sequences under the constraints of the non-selective steps in a SELEX experiment, we carried out a novel experiment called Neutral SELEX. In contrast to SELEX, Neutral SELEX does not select for any particular activity of interest, but includes only the amplification steps in the SELEX selection cycle, as shown by the red arrow in Figure 1. Differences in the resulting sequences from the library sequences should reflect requirements for survival in the neutral amplification steps.

We performed 10 rounds of Neutral SELEX, each round consisting of T7 transcription into RNA, followed by reverse transcription, and PCR. We began the experiment with the genomic *E. coli* library from the Hfq Genomic SELEX experiment, providing a basis for comparison to the positively selected RNAs. The initial library and the pools after each round were *454* sequenced, labeled 1 through 10 in the figures. We also sequenced the pool from the 9^th^ cycle of the Genomic SELEX for the selection of genomic aptamers binding the *E. coli* protein Hfq, which is labeled “H9”. The set of full-length *454* sequences (i.e. sequences with both primers) comprised the vast majority (170,284 of the total 191,577), therefore, we decided to analyze only these and omitted reads with only one primer. For all of the *in silico* analyses, we eliminated all primer and tag sequences.

**Figure 2:**
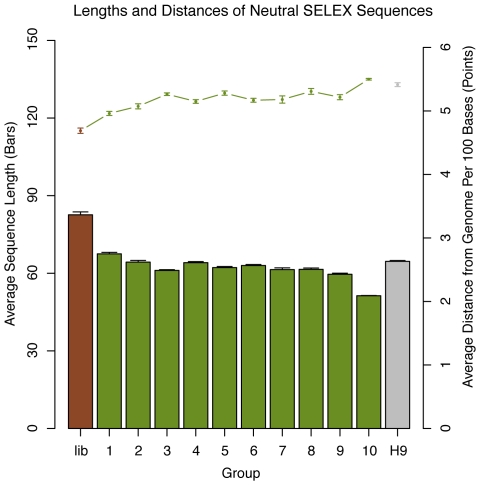
Effects of Neutral SELEX on length and distance to genomic sequence. The average lengths of the sequences in each Neutral SELEX pool, shown in bars, decreased dramatically after the library was transcribed and reverse transcribed into the first round of Neutral SELEX (left two bars), but only steadily thereafter. The average nucleotide became slightly more distant from the genomic sequence, shown in points and lines. The grey bar and the last point to the right indicate the average length and distance from the genome in the 9th round of the Hfq Genomic SELEX experiment.

We first looked at the lengths of the sequences, which are illustrated in Figure 2. It is important to note that the *454* machine that was used can sequence only about 250 bp per read, whereas the genomic library was intended to have inserts ranging from 50 to 500 bp. However, since 89% of the sequences were full length, it would seem the estimates of average length are valid. The average sequence length dropped sharply from 83 bases to about 68 upon initial transcription and reverse transcription (library to first round), but only steadily declines for the remainder of the rounds, with the 9th round at approximately 60 bp. H9 sequences are 65 bp long on average, slightly longer than the Neutral SELEX round 9 average.

In order to estimate the rate at which sequences mutate due to the amplification steps of SELEX, we next determined the distance of each pool's sequences from the genome, in terms of gaps and mismatches in the best alignment to the genome. We aligned each sequence to the *E. coli* K-12 genome using vmatch [13], taking the best alignment found with an E-value of less than 1e-10, if any. Each mismatch and gap was counted as an error, and the average distance was computed as the number of errors divided by the total number of aligned bases. Shown in Figure 2, the average percentage of erroneous bases increases only slightly with each successive round of Neutral SELEX.

It has been previously shown that the sequencing error rate of *454* machines is less than 1%, and that no correlation between length and error rate was observed when the sequences were not extremely long or short [14]. Since all pools were sequenced in one run, we can assume that mutations are induced by PCR, reverse transcription and T7 polymerase. However, the 0.9% change in error rate from the library to the 10th round of Neutral SELEX shown in Figure 2 is not very high.

### Changes in Structural Stability

To compare the predicted RNA secondary structural stability of the Hfq-selected sequences and the Neutral SELEX sequences, we used the Minimum Free Energy (MFE) *Z*-scores of each sequence to determine an average *Z*-score for each round of Neutral SELEX, the positively selected Hfq pool H9 and the sequenced genomic library. A *Z*-score compares the predicted MFE of the initial sequence to the distribution of several (in our case, 1000) randomized sequences of the same length and nucleotide content. The *Z*-score is precisely the difference, in number of standard deviations, between the original sequence and the mean MFE of the randomized sequences. Thus a low (negative) *Z*-score reflects a high structural stability contributed from the order of the sequence, and not the nucleotide content.

**Figure 3:**
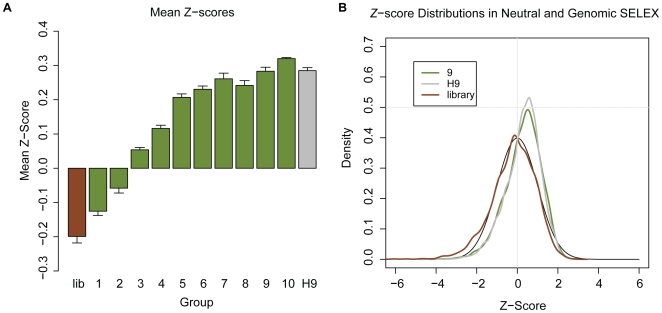
Effects of SELEX on structural stability of RNA sequences. (*A*) Average *Z*-score of sequences in the initial library (brown bar), each pool of Neutral SELEX (green bars) and the sequences from the Hfq Genomic SELEX experiment after 9 rounds of selection (grey bar). Numbers indicate the SELEX cycle. (*B*) Comparison of the distributions of *Z*-scores of the 9th round of Neutral SELEX, the 9th round of Hfq Genomic SELEX and the genomic library. These are all plotted next to the normal distribution (expected from random sequence), shown with the black line.

As shown in Figure 3A, the initial library has a negative average *Z*-score, which differs from the Hfq average on the right. With each successive round of Neutral SELEX, the average MFE *Z*-score increases, and the average *Z*-score becomes positive after the 3rd round, and ultimately approaches the average of the Hfq pool. This is a clear indication that the SELEX process favors sequences that are less structurally stable.

The average *Z*-score after 9 rounds of Neutral SELEX is nearly the same as the average after 9 rounds of Hfq SELEX, indicating that the SELEX process accounts for the positive *Z*-scores of the Hfq sequences. We also compared the *Z*-score distributions of these pools, shown in Figure 3B. The distribution of *Z*-scores is similar in the Hfq pool and the pool from round 9 of the Neutral SELEX experiment, further supporting the hypothesis that the neutral amplification steps of SELEX, not Hfq, imposed a requirement of structural instability on the sequences.

**Figure 4:**
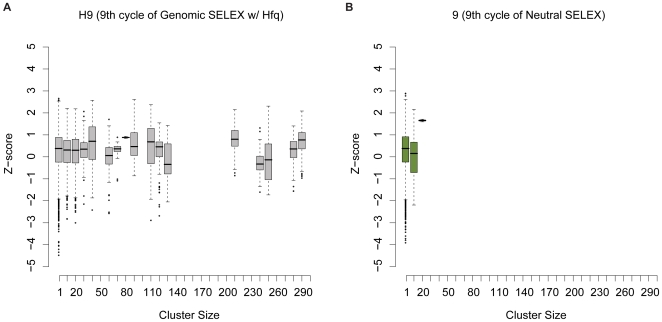
Enrichment effects on positively selected sequences. (*A*) Enrichment level plotted against *Z*-scores. Sequences selected by Genomic SELEX for Hfq were clustered if the best alignment shows mutual identity ≥85%. The cluster size is shown along the *x*-axis, and the *Z*-score of the sequence along the *y*-axis. The sequences were binned into cluster size ranges of 10, and the boxes represent the distribution of *Z*-scores within the range of cluster sizes. The boxes cover the 25%–75% range of the data, the line within the box is the median and the whiskers indicate 1.5× the interquartile range. If enrichment were dependent on a high *Z*-score, we would expect to see an increase in the median *Z*-score as cluster sizes increase, however this is not the case. In fact, the *Z*-score of any given sequence appears to vary nearly as much with enriched sequences as with unenriched sequences. (*B*) As a control, we plotted the same analysis with round 9 of Neutral SELEX, showing that enrichment is a signal of the positive selection of sequences.

This might also indicate that a sequence must be structurally unstable in order to be enriched by SELEX. In order to determine if this is the case, we estimated the enrichment level of each sequence by clustering the pool of positively selected Hfq sequences based on mutual sequence identity. Each sequence's enrichment was measured as the number of sequences in the cluster to which it belonged. We visualized the relationship between enrichment and stability in a box plot shown in Figure 4A. The *Z*-scores of the sequences did not substantially increase with enrichment level, and furthermore the variation of *Z*-score is similar between the enriched and non-enriched sequences. Therefore, it seems that SELEX favors structurally unstable sequences in general, independent of the positive selection.

We additionally measured the enrichment of the Neutral SELEX sequences after the 9th round using the same analysis (Figure 4B). As expected, no large clusters were found in the Neutral pool. Since no sequence specificity would be expected in selection through the neutral amplification steps, this also gives an estimate of the maximum size of clusters that appear by chance. 2099 of the 9991 sequences in the H9 pool belong to clusters with more than 50 sequences. The largest cluster in the Neutral pool has 16 sequences, showing that positive selection provides a clear signal of enrichment above the background.

### Nucleotide Content

**Figure 5:**
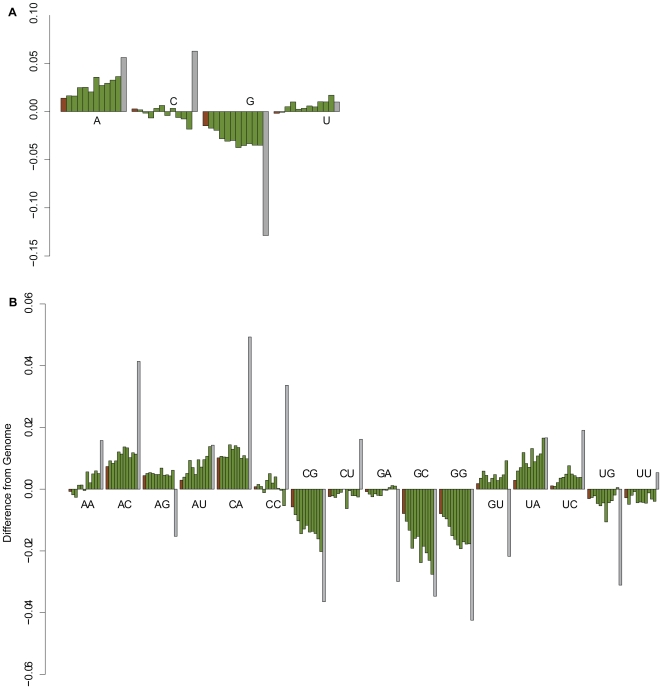
Trends in nucleotide frequencies of the Neutral SELEX and Hfq SELEX pools. Each group in the bar charts shows the difference in mononucleotide frequency (*A*) and dinucleotide frequency (*B*) from the averages of the *E. coli* K-12 genome. The groups begin on the left with the sequenced library in brown, then ten rounds of Neutral SELEX in green, and finally the Hfq Genomic SELEX content in grey.

We were also interested to see if SELEX amplification steps also influence the base composition. Since dinucleotide content influences the folding stability of genomic sequences [4], [15], [16], we were interested in both mono- and dinucleotide content. We measured the nucleotide content in each pool and compared this to the average content of the *E. coli* genome (Figure 5). In theory, the library content should be identical to that of the genome. However, the library content already shows a difference from the *E. coli* base composition, substantially contributing to the Neutral SELEX differences. Assuming the genomic sequence corresponds to the genome used experimentally, the library construction process must cause this difference. By visual comparison of Figure 5A along the ten rounds, we see a clear trend of increasing A and decreasing G content, and a weak trend of increasing U content. C content remains relatively stable. The chart in Figure 5B shows substantial trend of increasing AA, AU, GU, and UA dinucleotides and a substantial trend of decreasing in CG, GC and GG dinucleotides. Thus, the Neutral SELEX sequences show clear trends.

However, the grey bars in Figure 5 reveal remarkably different trends of the nucleotide content in the Hfq SELEX experiment. Thus, the amplification steps do not appear to impose requirements on the sequences that hamper selection for nucleotides that may be responsible for the binding activity of the enriched species.

## Discussion

Three popular options for the detection of novel, functional non-coding RNAs are RNomics, *in silico* screening and SELEX. RNomics (or cDNA sequencing) gives compelling evidence of an active RNA molecule, however, recovering RNAs is only possible when the sequence is abundantly transcribed. *In silico* screens are inexpensive, but are built upon known properties of RNA genes, begging the question of how to discover completely new trends. SELEX requires neither expression conditions nor a prior model, and thus provides a strong *in vitro* alternative to the constraints inherent in these screens. Where SELEX was, in the pre-genomic era, a tool to analyze the structure of ligand-binding RNAs or DNAs, the use of genomic sequences and pyrosequencing in Genomic SELEX transforms it into a tool to discover novel, functional domains of genomically encoded RNAs.

Researchers have raised concerns regarding the potential for biases imposed by the amplification steps both in print [11] and informally, but to our knowledge, there has been no attempt to make an estimate of the precise extent of these effects. Here we present the first data set sufficient to perform such an analysis. In analyzing our Neutral SELEX sequences, we pinpointed requirements imposed on the selected sequences, namely, the amplification steps favor sequences of lower structural stability, shorter sequences and (more weakly) sequences of some specific nucleotide content. These mostly derive from selectivity rather than mutational changes, as we saw negligible levels of sequence mutation due to PCR. We also showed that the distinction between binding sequences and neutrally selected sequences is much stronger in terms of nucleotide content and enrichment.

Along the way, we obtained sequences that can be useful in distinguishing signal from noise in the final enriched pool. Specifically, it can be useful to sequence the genomic library alongside the final pool. This can be especially important when dealing with repetitive genomes, because determining enrichment of a repetitive sequence is dependent on the copy number of the genome used, as well as any biases from whatever library construction method is employed. Sequences from a parallel Neutral SELEX experiment can also be useful in determining positional enrichment, e.g. the sequence surrounding translation start and stop signals [12], as well as enrichment of a motif, since there seems to be a slight bias in nucleotide content of the neutrally selected sequences.

Similar phenomena have been observed by Meyers and colleagues in a survey of SELEX-derived aptamers as compared to naturally occurring ones [11], as well as by Hallegger and colleagues, where SELEX was performed to detect RNA structural motifs with affinity to RNA editing factor ADAR, which binds dsRNAs, but no perfect double-stranded RNAs were sequenced from the resulting pool [17]. The authors were nevertheless able to detect structurally stable motifs among the recovered sequences, even if they do not represent the true, dominant binding motif. On the other hand, other SELEX experiments have resulted in the enrichment of highly structured and stable aptamers, which bind their respective ligands with high affinity [18]. Interestingly and in accordance with our observation, several *in vitro* selected aptamers were applicable as synthetic riboswitches, probably due to their low structural stability in the absence of the ligand. Both neomycin- and tetracycline-aptamers could be introduced into the 5′ UTR of eukaryotic genes, without impeding translation in the absence of the ligand, but undergoing allosteric changes and down regulating translation in the presence of the ligands [19], [20].

Our data extensively quantify the effects of SELEX on the evolution of sequences. We have shown that SELEX will have difficulty enriching some stable sequences. Libraries of Genomic SELEX, however, have lower complexity than those of random SELEX, making a strong, natural binder much more likely to be enriched. The genomic context of the genomic aptamers enriched, for example nearby transcripts, histone marks, or repeats, can also provide further clues about the candidates' functions. One can envision many applications of Genomic SELEX as a complementary approach to *in vivo* and *in silico* methods. We are confident that Genomic SELEX will be used extensively to determine the RNA regulatory network in cells, by identification of all regulatory RNA aptamers in RNA binding proteins involved in chromatin remodeling, transcription, posttranscriptional processing like alternative splicing, editing and localization as well as translation regulation.

## Materials and Methods

### Quality Assessment of the *E. coli* Genomic Library

All *454*-sequenced reads from the genomic library and other pools were assigned to regions in the K-12 genome by taking the best alignment found by vmatch [13]. Alignments were made by finding the best complete alignment diverging from the genomic sequence by no more than 80% of the bases. To determine the best alignment, we used our own scoring scheme, *S* = *M*−2(*MM*+*G*)/*L*, where *M* is the number of matches, *MM*, mismatches, *G*, gaps and *L* is the length of the query sequence. We measured the coverage of the library by dividing the total genomic bases covered by alignments by the total number of bases in the genome. The coverage of the genomic library found was 4.33%. We then wanted to know, given the amount of the sequence data obtained, how much coverage is expected from a library amplified from truly random genomic regions. This was done by generating *in silico* randomized libraries by repositioning every read to a random point in the genome. This was repeated 10,000 times, and the coverage of each randomized library was measured as before. The distribution had a minimum coverage of 5.32% and maximum coverage of 5.42%, and a mean of 5.37±1e-6%. From these sets the mean coverage divided by the coverage of the sequences library results in a redundancy rate of 25%.

### Neutral SELEX Experiment

The Genomic SELEX method, on which the Neutral SELEX method is based, has been described in detail [3]. The primers used were fixFORT7 5′ - CCAAGTAATACGACTCACTATAGGGGAATTCGGAGCGGG - 3′ and fixREV 5′ - CGGGATCCTCGGGGCTG - 3′. The T7 sequence comprises the first 21 nucleotides on the 5′ end of fixFORT7. We used a library originally generated for an Hfq Genomic SELEX experiment, generated as described in the protocol [3]. The DNA library was re-amplified with Fermentas Pfu polymerase in 10 cycles. Optimal yield was achieved with 3 µM MgSO_4_. Transcription of the library and each successive pool was carried out with NEB T7 polymerase, incubating for 2 hours, and performing the standard deactivation and NEB DNaseI treatment. Prior to setting up RT-PCR experiments, RNA was incubated for 5 minutes at 95°C and cooled on ice for at least one minute before adding to the reaction. RT-PCR was done with the QIAGEN One-Step kit, using 10 cycles and otherwise using the standard protocol without Q solution.

The sequences were prepared for *454* sequencing using the following primers: fixFor_454_Tag 5′ - GCCTCCCTCGCGCCATCAGNNAGGGGAATTCGGAGCGGG - 3′ and fixRev_454_Tag 5′ - GCCTTGCCAGCCCGCTCAGNNCGGGATCCTCGGGGCTG - 3′. In both of these primers, the *454* adaptors comprise the first 19 nucleotides from the 5′ end, followed by the “NN” sequence tag, and all nucleotides 3′ of this tag come from the fixFOR and fixREV primers of SELEX. All 10 rounds of Neutral SELEX, as well as the Genomic SELEX library, and round 9 of the Hfq SELEX experiment were sequenced, and the “NN” sequence is a tag indicating to which of these pools the sequence belongs. In order to get sufficient yields, two rounds of Pfu PCR were performed with 8 cycles each. The samples were sent to Vertis Biotech for primer removal, mixing and *454* sequencing.

### Filtering of *454* Sequences

The 191,577 sequences obtained from *454* sequencing were annotated using a script that employs vmatch [13]. All sequences were constrained to have an exact match of the fixFOR and fixREV primer, and a matching bucket tag on both ends. The sequences used for analysis had no primers or bucket tags. The final pool contained 170,284 sequences.

### Genomic Analysis of Neutral SELEX Results

The *E. coli* K-12 genome was downloaded from GenBank [21] (accession NC_000913). The distance from the genome was determined with genomic alignments using vmatch, as described in the Quality Assessment section above. Distance was calculated as the total number of mismatches and gaps in the alignment divided by the length of the query (*454*) sequence.

Sequences were clustered using the vmatch program -dbcluster option, requiring clustered sequences to show mutual 85% identity, and the alignments be at least 20 bases long.

### *Z*-Score Calculations of SELEX Results

To calculate *Z*-scores, the sequences were folded using RNAfold from the ViennaRNA package version 1.7.2 [22], using default parameters. Sequences were also shuffled 1000 times preserving dinucleotide content as described by Altschul and Erickson [23] and implemented in the squid library [Eddy, unpublished]. For each sequence *i* we computed normalized *Z*-scores of the minimum free energy *m*(*i*), with *z*(*i*) = (*m*(*i*)−μ(*i*))/σ(*i*), where the mean μ(*i*) and the standard deviation σ(*i*) are calculated from the shuffled sequences of *i* respectively. In cases where all predicted free energies of the folding sequences were exactly identical the standard deviation could be 0, making the *Z*-score undefined. This occurs when no base pairing was possible, for example in short, homogeneous sequences. We eliminated these sequences from analysis, since they occurred rarely, in only 625 of the 170,284 sequences.

The distribution of these *Z*-scores, and all other numeric-based charts in these figures, were visualized using the R package [24].
